# Ciprofloxacin Versus Fosfomycin for Empirical Prophylaxis Before Transrectal Prostate Biopsy: Clinical, Microbiological, and Patient-Reported Outcomes from a Prospective Study

**DOI:** 10.3390/medsci14010091

**Published:** 2026-02-14

**Authors:** Edgaras Burzinskis, Ieva Janulaityte, Guoda Burzinskiene, Darijus Skaudickas, Albinas Naudziunas, Astra Vitkauskiene

**Affiliations:** 1 Department of Laboratory Medicine, Lithuanian University of Health Sciences, 44307 Kaunas, Lithuania; ieva.janulaityte@lsmu.lt (I.J.); astra.vitkauskiene@lsmuni.lt (A.V.); 2 Department of Obstetrics and Gynecology, Lithuanian University of Health Sciences, 44307 Kaunas, Lithuania; guoda.juskeviciute@lsmu.lt; 3 Department of Urology, Lithuanian University of Health Sciences, 44307 Kaunas, Lithuania; darijus.skaudickas@lsmu.lt; 4 Department of Internal Medicine, Lithuanian University of Health Sciences, 44307 Kaunas, Lithuania; albinas.naudziunas@lsmu.lt

**Keywords:** prostate biopsy, antibiotic prophylaxis, ciprofloxacin, fosfomycin, antimicrobial resistance, targeted prophylaxis, quality of life

## Abstract

Background: Transrectal ultrasound-guided prostate biopsy remains the gold standard in diagnosing prostate cancer, but is associated with infectious and non-infectious complications. Increasing fluoroquinolone resistance and regulatory restrictions have prompted evaluation of alternative prophylactic strategies, including fluoroquinolone-sparing agents and targeted prophylaxis. This study compared ciprofloxacin and fosfomycin as empirical prophylactic agents, focusing on microbiological concordance, clinical outcomes, and patient-reported outcomes. Methods: In this prospective observational study, 265 men undergoing transrectal ultrasound-guided prostate biopsy received empirical antibiotic prophylaxis with either ciprofloxacin (*n* = 146) or fosfomycin trometamol (*n* = 119). Rectal swabs were obtained prior to biopsy, and antimicrobial susceptibility was analyzed post hoc. Infectious and non-infectious complications were recorded. Lower urinary tract symptoms (IPSS), erectile function (IIEF-5), and patient-reported quality of life were assessed before and after biopsy. Results: Microbiological concordance between administered prophylaxis and rectal flora susceptibility was higher in the ciprofloxacin group than in the fosfomycin group (80.1% vs. 65.0%, *p* = 0.007), while resistance rates were similar (10.9% vs. 10.2%). Post-biopsy fever occurred in 5.3% of patients, and hospitalization was required in 3.1%, with no significant differences between prophylaxis groups. IPSS increased significantly after biopsy (*p* < 0.001), while IIEF-5 scores remained unchanged. Patients with microbiological concordance reported significantly better post-biopsy quality of life (*p* = 0.006). Conclusions: Ciprofloxacin and fosfomycin showed similar safety profiles as empirical prophylaxis before transrectal prostate biopsy. Although ciprofloxacin achieved higher microbiological concordance, fosfomycin remains a viable alternative. The link between microbial concordance and improved patient-reported quality of life underscores the importance of targeted prophylaxis and supports antimicrobial stewardship in prostate cancer diagnostics.

## 1. Introduction

Prostate cancer (PCa) is among the most prevalent malignancies in men worldwide and remains a major contributor to cancer-related mortality [1]. Transrectal ultrasound-guided biopsy is central to diagnosis but is associated with clinically relevant infectious complications. The same trends are seen in the United States, where lifetime risk estimates indicate that about 16% of men will be diagnosed with prostate cancer [2]. Early-stage disease is frequently associated with favorable survival outcomes, but a significant number of patients experience disease progression, biochemical relapse, or resistance to treatment. Additionally, prostate cancer still accounts for a significant number of cancer-related deaths, with roughly one in every thirty diagnosed individuals eventually succumbing to the disease.

Given these considerations, diagnostic strategies that prioritize accuracy while reducing patient harm are essential. TRUS-guided prostate biopsy remains a gold standard of prostate cancer diagnostics and is routinely performed worldwide, with annual procedure numbers exceeding one million [3,4]. Despite its widespread use, this intervention is not without risk. Post-biopsy infectious complications range from mild febrile illness to severe, life-threatening sepsis requiring hospital admission, representing a clinically meaningful source of morbidity [3,4,5].

For decades, fluoroquinolones (especially Ciprofloxacin) have been the preferred antibiotics for antimicrobial prophylaxis prior to transrectal prostate biopsy. This preference has primarily been driven by favorable oral bioavailability and effective penetration into prostatic tissue [4,6]. However, increasing rates of fluoroquinolone-resistant rectal flora have been documented globally and are strongly associated with rising post-biopsy infection rates [3,5,7]. In addition, safety concerns and regulatory warnings have restricted fluoroquinolone use in several regions, further limiting their suitability as routine prophylactic agents and highlighting the need for alternative approaches [8].

Rectal swab-guided targeted antibiotic prophylaxis has emerged as one such alternative, individualizing antimicrobial selection based on pre-biopsy microbiological assessment of rectal flora. This strategy has consistently been shown to reduce infectious complications when compared with empirical prophylaxis [5,7]. Meta-analyses involving large patient cohorts indicate that targeted prophylaxis significantly reduces post-biopsy infection rates [7]. Adherence to guideline-recommended prophylactic strategies has been identified as a key component of antimicrobial stewardship in urological practice [9]. Despite these advantages, routine implementation remains limited by logistical challenges, additional costs, and delays related to microbial testing, leading to continued widespread use of empirical prophylaxis.

In this context, fosfomycin trometamol has emerged as a promising empirical alternative. Fosfomycin demonstrates broad antimicrobial activity against Gram-negative and Gram-positive organisms, including multidrug-resistant strains. Following oral administration, fosfomycin attains therapeutically relevant concentrations in prostatic tissue, supporting its pharmacokinetic suitability for prophylactic use in transrectal prostate biopsy [10,11,12]. Clinical trials and systematic reviews have reported infection rates comparable to those observed with fluoroquinolone prophylaxis, supporting its role as a fluoroquinolone-sparing option aligned with antimicrobial stewardship principles [3,10,11].

Beyond infectious complications, transrectal prostate biopsy is associated with non-infectious adverse events such as hematuria, hematospermia, and transient exacerbation of lower urinary tract symptoms (LUTS), all of which may negatively affect patient quality of life [4,13]. Acute bacterial prostatitis following biopsy has been linked to prolonged symptom duration and increased patient discomfort [13]. Nevertheless, patient-reported outcomes, including urinary symptoms, sexual function, and health-related quality of life, are infrequently incorporated into comparative assessments of prophylactic strategies, particularly in relation to microbiological concordance between administered antibiotics and rectal flora susceptibility.

The present prospective observational study was designed to compare ciprofloxacin and fosfomycin as empirical prophylactic antibiotics before TRUS-guided prostate biopsy. In addition to evaluating microbiological susceptibility concordance and rates of infectious and non-infectious complications, this study emphasizes patient-reported outcomes. It explores whether alignment between prophylactic antibiotic choice and rectal flora susceptibility influences post-biopsy symptom burden and quality of life.

## 2. Materials and Methods

### 2.1. Study Design and Setting

This prospective observational study was conducted in Lithuania at the Department of Laboratory Medicine and the Hospital of the Lithuanian University of Health Sciences (LUHS). Consecutive male patients referred for TRUS-guided prostate biopsy due to suspected prostate cancer were enrolled. The study was approved by the Kaunas Regional Biomedical Research Ethics Committee (approval No. BE-2-10, 22 March 2024) and written consents from all subjects were received.

The study followed the STROBE guidelines for observational studies. The study reflects routine clinical practice within a structured national prostate cancer diagnostic framework, in which patients with elevated prostate-specific antigen levels and/or abnormal digital rectal examination findings are referred for urological evaluation and prostate biopsy when clinically indicated.

### 2.2. Patient Selection

Eligible participants were adult men (≥18 years) undergoing TRUS-guided prostate biopsy for suspected prostate cancer. Indications for biopsy included elevated prostate-specific antigen levels, abnormal digital rectal examination, or other clinical findings suggestive of malignancy. All patients received empirical antibiotic prophylaxis prior to biopsy.

Exclusion criteria included evidence of active urinary tract infection at the time of biopsy, systemic antibiotic therapy within four weeks prior to the procedure, known immunodeficiency, current use of immunosuppressive therapy, or incomplete clinical, microbiological, or patient-reported outcome data.

### 2.3. Biopsy Procedure

All prostate biopsies were performed using a standardized transrectal ultrasound-guided technique with an 18-gauge biopsy needle. A systematic 12-core biopsy scheme was applied in all patients. Local anesthesia was administered according to institutional protocols.

To ensure procedural consistency and minimize operator-related variability, all biopsies were performed by a single experienced urologist following principles of good clinical practice.

### 2.4. Antibiotic Prophylaxis

Empirical antibiotic prophylaxis was administered at the Lithuanian University of Health Sciences Kaunas Hospital in accordance with local institutional protocol for transrectal prostate biopsy. Patients were randomly assigned to receive either ciprofloxacin or fosfomycin trometamol. Ciprofloxacin was administered orally at a dose of 500 mg twice daily, starting one day before biopsy and continued for a total of 5 days. Fosfomycin trometamol was administered orally as a 3 g dose the evening before biopsy and repeated 24 h after the procedure. The choice of antibiotic was empirical and not guided by rectal swab culture results at the time of biopsy. This approach minimized selection bias and allowed comparison of clinical and microbiological outcomes between empirically prescribed prophylactic regimens.

### 2.5. Rectal Swab Sampling and Microbiological Analysis

Rectal swab samples were obtained from all patients prior to biopsy and processed using standard microbiological culture techniques. Antimicrobial susceptibility testing of rectal swab isolates was performed according to the European Committee on Antimicrobial Susceptibility Testing (EUCAST) clinical breakpoints valid at the time of analysis. Isolates were categorized as susceptible, intermediate, or resistant. For the purposes of this study, isolates classified as resistant were considered microbiologically non-susceptible to the administered prophylactic antibiotic.

Microbiological concordance was operationally defined as susceptibility of all isolated organisms to the empirically administered prophylactic antibiotic, whereas microbiological discordance was defined as resistance of at least one isolated organism to the administered agent.

Of the 265 enrolled patients, microbiological culture data were unavailable in 12 cases (4.5%) due to insufficient sample yield or inconclusive susceptibility testing; these cases were excluded from concordance analyses. Microbiological results were analyzed retrospectively and did not influence antibiotic selection at the time of biopsy.

### 2.6. Outcome Assessment

Post-biopsy outcomes were assessed through standardized clinical follow-up and patient-reported questionnaires. Infectious complications were defined as post-biopsy fever and/or hospitalization due to infection. Non-infectious complications included hematuria and hematospermia.

Patient-reported outcomes were assessed at two predefined time points: at baseline prior to biopsy and at a standardized follow-up 14 days after the procedure. Lower urinary tract symptoms were evaluated using the International Prostate Symptom Score (IPSS), and erectile function was assessed using the five-item International Index of Erectile Function (IIEF-5). Quality of life was measured using the single-item quality-of-life question from the IPSS (score range 0–6).

The severity and duration of hematuria and hematospermia were recorded based on patient self-report, with patients indicating the number of days symptoms persisted following biopsy. These outcomes were analyzed as continuous variables reflecting symptom duration (in days), rather than using a formal ordinal grading scale.

Histopathological findings, including the presence of chronic prostatitis, were documented and included in predefined subgroup analyses.

### 2.7. Statistical Analysis

Statistical analyses were performed using SPSS Statistics version 29.0.1 (IBM Corp., Armonk, NY, USA). Continuous variables are presented as means with standard deviations or medians with interquartile ranges, as appropriate. Categorical variables are presented as frequencies and percentages.

Between-group comparisons were conducted using the Mann–Whitney U test for continuous variables and the chi-square test for categorical variables. Paired pre- and post-biopsy comparisons were assessed using the Wilcoxon signed-rank test. A two-sided *p*-value of <0.05 was considered statistically significant.

### 2.8. Ethical Considerations

The study was conducted in accordance with the Declaration of Helsinki and approved by the Kaunas Regional Biomedical Research Ethics Committee (approval number BE-2-10; approval date 22 March 2024). Written informed consent was obtained from all participants prior to their inclusion in the study.

## 3. Results

### 3.1. Study Population and Baseline Characteristics

A total of 265 men underwent TRUS-guided prostate biopsy and were included in the analysis. Empirical antibiotic prophylaxis consisted of ciprofloxacin in 55.1% (*n* = 146) and fosfomycin in 44.9% (*n* = 119) of cases. Baseline demographic and clinical characteristics are summarized in Table 1. The median age did not differ significantly between groups, and baseline IPSS, erectile function scores, and quality-of-life (QoL) assessments were comparable.

Vast majority of microbiological cultures were available for analysis in the study cohort.

Rectal swab cultures yielded bacterial growth in 262 of 265 patients (98.9%). The most frequently isolated organism was *Escherichia coli* (77.1%), followed by other *Enterobacterales*, including *Klebsiella* spp., *Enterobacter* spp., *Citrobacter* spp., and *Proteus* spp. (13.4%). *Enterococcus* spp. accounted for 5.3% of isolates, while streptococcal species, including beta-hemolytic streptococci, were identified in 4.2% of cases. No growth or inconclusive cultures were observed in 1.1% of patients.

Extended-spectrum beta-lactamase (ESBL) production and resistance to non-study antibiotics were not systematically analyzed in this cohort. Therefore, the prevalence of highly resistant organisms could not be reliably assessed.

Overall, microbiological discordance between the empirically administered prophylactic antibiotic and rectal flora susceptibility was identified in 26.9% of cases, whereas microbiological concordance was observed in 73.1% of patients.

Ciprofloxacin and fosfomycin resistance rates were comparable (10.9% and 10.2%, respectively) and did not differ significantly according to the number of isolated microorganisms (monomicrobial vs. polymicrobial cultures). Patient numbers vary across analyses due to missing questionnaire data or because certain outcomes (e.g., symptom duration, fever-related outcomes) applied only to subsets of patients.

### 3.2. Overall Effect of Prostate Biopsy on Patient-Reported Outcomes

The overall impact of prostate biopsy on patient-reported outcomes is presented in Table 2. Lower urinary tract symptoms worsened significantly after biopsy, with IPSS scores increasing after the procedure (*p* < 0.001). Although most patients experienced no change in IPSS, a substantial subset showed worsening of symptoms, while improvement occurred infrequently.

Erectile function, assessed using the IIEF-5 questionnaire, did not change significantly following biopsy (*p* = 0.754), with the majority of patients demonstrating identical pre- and post-biopsy scores.

Quality-of-life scores showed a small but statistically significant deterioration after biopsy (*p* < 0.001), despite similar median values before and after the procedure.

### 3.3. Comparison of Outcomes by Empirical Antibiotic Prophylaxis

Clinical outcomes according to empirical antibiotic prophylaxis are summarized in Table 3. The incidence of post-biopsy fever did not differ significantly between patients receiving ciprofloxacin and those receiving fosfomycin (5.6% vs. 5.1%; *p* = 0.866). Rates of hospitalization, medical consultation after biopsy, chills, and worsening of lower urinary tract symptoms were also comparable between prophylaxis groups (all *p* > 0.05).

Post-biopsy infectious complications included fever and chills, and a small proportion of patients required hospitalization and intravenous antibiotic therapy. These events were managed as severe systemic infections; however, no cases progressed to septic shock or required intensive care unit admission.

Hematuria and hematospermia severity did not differ significantly between antibiotic groups (*p* = 0.262 and *p* = 0.118, respectively).

Post-biopsy IPSS scores were significantly higher among patients receiving ciprofloxacin compared with fosfomycin (*p* = 0.032). Post-biopsy QoL scores tended to be worse in the ciprofloxacin group; however, this difference did not reach statistical significance (*p* = 0.055). Post-biopsy erectile function scores were similar between antibiotic groups (*p* = 0.266).

### 3.4. Microbiological Concordance of Empirical Prophylaxis

The associations between microbiological concordance of empirical prophylaxis and patient-reported and clinical outcomes are summarized in Table 4.

Microbiological discordance between empirical prophylaxis and rectal flora susceptibility was significantly more frequent in patients receiving fosfomycin than in those receiving ciprofloxacin (35.0% vs. 19.9%; *p* = 0.007).

Baseline and post-biopsy IPSS scores did not differ significantly between patients with microbiologically concordant versus discordant prophylaxis (*p* = 0.807 and *p* = 0.709, respectively). The severity and duration of hematuria and hematospermia were also comparable between concordant and discordant groups.

In contrast, post-biopsy quality-of-life scores were significantly worse among patients with microbiologically discordant prophylaxis (*p* = 0.006), whereas post-biopsy IPSS severity and erectile function scores did not differ significantly according to microbiological concordance.

Microbiological discordance was significantly more frequent among patients receiving fosfomycin than among those receiving ciprofloxacin (35.0% vs. 19.9%; *p* = 0.007).

Baseline and post-biopsy IPSS scores did not differ significantly between patients with microbiologically concordant and discordant empirical prophylaxis (*p* = 0.807 and *p* = 0.709, respectively). Hematuria and hematospermia severity were likewise comparable between concordant and discordant groups.

Post-biopsy quality-of-life scores were significantly worse in patients with microbiologically discordant prophylaxis (*p* = 0.006), whereas post-biopsy IPSS severity and erectile function scores did not differ significantly according to microbiological concordance.

### 3.5. Impact of Post-Biopsy Fever

Post-biopsy fever occurred in 5.3% of patients and was strongly associated with symptom burden and deterioration in quality of life. QoL worsening was observed in 92.9% of patients who developed fever compared with 4.0% of those without fever (χ^2^ = 130.6, *p* < 0.001).

Patients with post-biopsy fever had significantly higher post-biopsy IPSS scores than afebrile patients (median 20.5 [range 8–35] vs. 9.0 [range 0–32]; *p* < 0.001) and reported significantly worse QoL (median 4.5 [range 2–6] vs. 1.0 [range 0–6]; *p* < 0.001). Post-biopsy erectile function scores did not differ significantly between febrile and afebrile patients.

### 3.6. Histopathological Findings

Patients with chronic prostatitis did not differ significantly from those with other histopathological findings (prostate cancer or benign prostatic hyperplasia) with respect to age, PSA levels, microbial growth pattern (monomicrobial versus polymicrobial), or antimicrobial resistance rates.

However, post-biopsy IPSS scores were significantly higher in patients with chronic prostatitis (median 11.0 vs. 9.0; *p* = 0.041), and post-biopsy erectile function scores were also higher in this group (median 15.0 vs. 10.0; *p* = 0.017). No significant differences were observed in post-biopsy QoL scores, hematuria, hematospermia, or duration of symptoms.

## 4. Discussion

In this prospective observational study, we compared ciprofloxacin and fosfomycin as empirical antibiotic prophylaxis before TRUS-guided prostate biopsy, integrating microbiological susceptibility data, clinical complications, and patient-reported outcomes. The most important findings indicate that, despite significantly higher microbiological concordance with ciprofloxacin, both prophylactic regimes were associated with similarly low rates of infectious (and non-infectious) complications. Significantly, concordance between the administered antibiotic and rectal flora susceptibility was associated with improved post-biopsy quality of life, highlighting a clinically meaningful benefit of concordant antimicrobial coverage that extends beyond the prevention of acute infection.

Notably, several outcomes reached statistical significance and warrant emphasis. Empirical ciprofloxacin prophylaxis demonstrated significantly higher microbiological concordance compared with fosfomycin (*p* = 0.007). Post-biopsy lower urinary tract symptoms were significantly more severe in patients receiving ciprofloxacin compared with fosfomycin (*p* = 0.032). Importantly, microbiological discordance between prophylaxis and rectal flora susceptibility was independently associated with significantly worse post-biopsy quality of life (*p* = 0.006). In addition, lower urinary tract symptoms worsened significantly after biopsy in the overall cohort (*p* < 0.001), confirming the transient symptomatic burden associated with the procedure.

Fluoroquinolones have historically been regarded as the standard prophylactic agents for transrectal prostate biopsy because of their favorable pharmacokinetic properties and effective penetration into prostatic tissue. However, a substantial body of evidence has demonstrated a global rise in fluoroquinolone-resistant rectal flora, which is now recognized as the dominant risk factor for post-biopsy infectious complications [3,4,5]. Extensive cohort studies and microbiological analyses consistently show that infections occurring despite fluoroquinolone prophylaxis are predominantly caused by fluoroquinolone-resistant *Escherichia coli*, underscoring the central role of prophylactic–microbiological discordance in biopsy-related morbidity [5].

In response to these trends, fosfomycin trometamol has emerged as a fluoroquinolone-sparing alternative. Fosfomycin demonstrates broad antimicrobial activity against Gram-negative uropathogens, including multidrug-resistant and extended-spectrum beta-lactamase-producing organisms, and achieves high urinary and fecal concentrations following oral administration [10,11,12]. Multiple comparative studies and systematic reviews have reported comparable rates of post-biopsy fever, sepsis, and hospitalization between fosfomycin-based and fluoroquinolone-based prophylaxis regimens [3,14]. Our findings are consistent with this literature, demonstrating similar clinical safety profiles for ciprofloxacin and fosfomycin despite differences in microbiological susceptibility concordance.

The lower concordance observed with fosfomycin in our cohort likely reflects local resistance ecology rather than inferior intrinsic antimicrobial efficacy. Notably, resistance rates to ciprofloxacin and fosfomycin were similar, reinforcing the concept that empirical prophylaxis, regardless of the antimicrobial agent used, cannot reliably ensure appropriate coverage in all patients. This observation aligns with pooled analyses indicating that empirical prophylaxis fails primarily due to unrecognized rectal colonization with resistant organisms rather than shortcomings of specific antibiotics [3,7].

Targeted prophylaxis guided by rectal swab cultures has been extensively evaluated as a strategy to overcome this limitation [15]. Several randomized trials and meta-analyses have demonstrated that targeted prophylaxis significantly reduces infectious complications compared with empirical regimens, particularly in settings with high fluoroquinolone resistance [3,7,16]. The benefits appear consistent across different antimicrobial classes, suggesting that the advantage lies in microbiological matching rather than antibiotic selection alone. Nevertheless, despite strong supporting evidence, routine implementation of targeted strategies remains limited by logistical challenges, economic constraints, laboratory infrastructure, and delays in culture result availability, resulting in continued reliance on empirical prophylaxis in everyday clinical practice.

Beyond fluoroquinolones and fosfomycin, alternative prophylactic strategies have been explored, including cephalosporins, aminoglycosides, combination regimens, and augmented prophylaxis in high-risk patients, as well as targeted prophylaxis guided by rectal swab cultures. Several studies have demonstrated reduced infectious complication rates with tailored antimicrobial regimens or combination approaches incorporating aminoglycosides in selected patients. In parallel, the increasing adoption of transperineal biopsy techniques has been proposed as a non-antibiotic strategy to minimize infectious risk by avoiding rectal flora translocation [15]. Nevertheless, transrectal biopsy remains widely practiced in many centers due to accessibility, cost considerations, and operator familiarity. Therefore, optimization of antimicrobial prophylaxis within this framework remains clinically relevant. Our findings contribute to this evolving landscape by supporting fosfomycin as a fluoroquinolone-sparing option while reinforcing the potential value of microbiologically informed prophylactic strategies.

A key strength of the present study is the inclusion of validated patient-reported outcome measures, an aspect that remains insufficiently addressed in prior research on prostate biopsy prophylaxis. Consistent with previous studies, we observed a significant increase in lower urinary tract symptoms following biopsy, reflecting the procedure’s transient inflammatory and mechanical impact [4]. Erectile function, assessed using the IIEF-5, remained stable, supporting earlier evidence that transrectal prostate biopsy has minimal short-term impact on sexual function. Importantly, patients who developed post-biopsy fever experienced a markedly higher symptom burden, emphasizing that even infrequent infectious complications can have disproportionate effects on patient comfort and recovery.

Of particular novelty, microbiological concordance between the administered prophylactic antibiotic and rectal flora susceptibility was associated with improved post-biopsy quality of life. While previous studies have primarily focused on infection prevention as the principal endpoint, our findings suggest that concordant antimicrobial coverage may influence broader patient experience, potentially by reducing subclinical infections, inflammatory responses, or psychological distress. This patient-centered observation strengthens the rationale for targeted prophylaxis strategies and aligns with emerging calls to incorporate quality-of-life outcomes into evaluations of prostate biopsy safety and the effectiveness of prophylaxis.

Although post-biopsy fever and hospitalization were numerically more frequent among patients with microbiologically concordant prophylaxis, these differences were not statistically significant. In contrast, overall post-biopsy quality of life was significantly worse among patients with microbiologically discordant prophylaxis, suggesting that patient-reported QoL may reflect a broader spectrum of post-biopsy symptom burden beyond overt febrile complications alone.

Given the relatively small number of infectious events, the study may have been underpowered to detect subtle differences in clinical complications between concordant and non-concordant groups. Larger, adequately powered cohorts are warranted to further clarify the relationship between microbiological concordance, infectious outcomes, and patient-reported quality of life.

From an antimicrobial stewardship perspective, the continued routine use of fluoroquinolones has become increasingly controversial. Regulatory authorities in Europe and elsewhere have issued warnings and restrictions regarding fluoroquinolone use due to concerns about serious adverse effects and ecological impact [8,17]. Fosfomycin offers several stewardship advantages, including a single-dose regimen, limited systemic exposure, and preserved activity against multidrug-resistant organisms [10]. However, increasing reliance on fosfomycin also necessitates careful surveillance to prevent the emergence of resistance, underscoring that stewardship principles must guide both empirical and targeted prophylactic strategies.

Although transperineal biopsy techniques have been proposed as a means to reduce infectious complications, transrectal prostate biopsy remains widely used worldwide because of accessibility, cost considerations, and operator familiarity. Consequently, optimization of prophylactic strategies for transrectal biopsy remains clinically relevant, particularly in regions where transperineal approaches are not yet universally adopted. In this context, our findings provide pragmatic evidence supporting both fluoroquinolone-sparing regimens and microbiologically informed prophylaxis strategies.

Several limitations should be acknowledged in this single-center-based study. The observational design limits causal inference, and antibiotic selection was based on empirical rather than randomized methods. The single-center design may limit generalizability to regions with different antimicrobial resistance patterns. Although rectal swabs were obtained from all patients, susceptibility data were analyzed post hoc and did not influence antibiotic selection at the time of biopsy. Finally, long-term outcomes beyond the early post-biopsy period were not assessed.

Although multivariable regression modeling could further clarify independent predictors of post-biopsy infectious complications and patient-reported outcomes, the relatively low event rates in this cohort limited the statistical power for robust adjusted analyses. Future larger multicenter studies will be required to confirm these associations and to identify independent risk factors in multivariable models.

Despite these limitations, the study is strengthened by its prospective design, standardized biopsy technique performed by a single urologist, comprehensive microbiological assessment, and integration of validated patient-reported outcome measures. These features enhance the internal consistency and clinical relevance of the findings.

## 5. Conclusions

Ciprofloxacin and fosfomycin demonstrated comparable clinical safety as empirical prophylaxis before transrectal prostate biopsy. Although ciprofloxacin achieved higher microbiological concordance, only concordance itself—not antibiotic class—was associated with improved quality of life. These findings support wider adoption of microbiologically guided prophylaxis within antimicrobial stewardship frameworks.

## Figures and Tables

**Table 1:** Baseline demographic and clinical characteristics of the study cohort.

Characteristic	Value, *n* (%)
Number of patients	265
Age, years, median (range)	66 (46–84)
PSA, ng/mL, median (range)	6.56 (0.58–600.0)
Empirical antibiotic prophylaxis	
Ciprofloxacin	146 (55.1%)
Fosfomycin	119 (44.9%)
Microbiological concordance of empirical prophylaxis	
Concordant	185 (73.1%)
Discordant	68 (26.9%)
Antibiotic resistance	
Ciprofloxacin-resistant isolates	29 (10.9%)
Fosfomycin-resistant isolates	27 (10.2%)
Number of isolated microorganisms	
One organism	144 (54.9%)
≥2 organisms	116 (44.3%)
Post-biopsy adverse events	
Fever	14 (5.3%)
Hospitalization	8 (3.1%)
Medical consultation after biopsy	11 (4.2%)
Chills	12 (4.6%)
Hematuria	193 (72.8%)
Hematospermia	55 (20.8%)

Abbreviations: PSA, prostate-specific antigen; Values are presented as *n* (%) unless otherwise stated. Medians are reported with minimum–maximum ranges. Microbiological concordance refers to agreement between empirical antibiotic prophylaxis and rectal swab antimicrobial susceptibility. “≥2 organisms” indicates polymicrobial growth on rectal swab culture. Patient numbers may vary across variables due to missing data or outcome-specific applicability.

**Table 2:** Paired comparison of patient-reported functional outcomes before and after transrectal prostate biopsy.

Outcome	Before Biopsy Median (Min–Max)	After Biopsy Median (Min–Max)	Test Statistic	*p* Value
Quality of life score	1 (0–6)	1 (0–6)	Z = −5.371	<0.001
IPSS score	8 (0–32)	9.5 (0–35)	Z = −8.146	<0.001
Erectile function score	11 (0–25)	11 (0–25)	Z = −0.313	0.754

Abbreviations: IPSS, International Prostate Symptom Score; Notes: Analyses were performed on paired pre- and post-biopsy data (*n* = 262).

**Table 3:** Post-biopsy clinical outcomes according to empirical antibiotic prophylaxis.

Outcome	Ciprofloxacin	Fosfomycin	Test Statistic	*p* Value
Hematuria, days, median (range)	2.5 (1–14)	2 (1–12)	Z = −1.121	0.262
Hematospermia, days, median (range)	14 (5–30)	10 (3–21)	Z = −1.565	0.118
Post-biopsy fever, *n* (%)	8/144 (5.6%)	6/118 (5.1%)	χ^2^ = 0.028	0.866
Hospitalization, *n* (%)	5/144 (3.5%)	3/118 (2.5%)	χ^2^ = 0.189	0.663
Medical consultation, *n* (%)	5/144 (3.5%)	6/118 (5.1%)	χ^2^ = 0.419	0.517
LUTS worsening, *n* (%)	19/144 (13.2%)	17/118 (14.4%)	χ^2^ = 0.080	0.777
IPSS after biopsy, median (range)	10 (0–32)	8.5 (0–35)	Z = −2.144	0.032
Post-biopsy QoL, median (range)	2 (0–6)	1 (0–6)	Z = −1.919	0.055
Erectile function after biopsy, median (range)	10 (0–25)	12 (0–25)	Z = −1.112	0.266

Abbreviations: IPSS, International Prostate Symptom Score; QoL, quality of life; LUTS, lower urinary tract symptoms. Notes: Analyses include patients with available post-biopsy data (ciprofloxacin, *n* = 144; fosfomycin, *n* = 118).

**Table 4:** Baseline characteristics and microbiological concordance according to empirical prophylaxis concordance.

Variable	Concordant Prophylaxis	Non-Concordant Prophylaxis	Test Statistic	*p* Value
IPSS before biopsy, median (range)	10 (0–28)	8 (0–32)	Z = −0.244	0.807
IPSS after biopsy, median (range)	10 (0–32)	9 (0–35)	Z = −0.373	0.709
Erectile function before biopsy, median (range)	14 (0–25)	10 (0–25)	Z = −1.699	0.089
Erectile function after biopsy, median (range)	13.5 (0–25)	10 (0–25)	Z = −1.738	0.082
Post-biopsy fever, *n* (%)	4 (6.1%)	10 (5.4%)	χ^2^ = 0.036	0.850
Hospitalization, *n* (%)	3 (4.5%)	5 (2.7%)	χ^2^ = 0.524	0.469
Medical consultation, *n* (%)	3 (4.5%)	8 (4.3%)	χ^2^ = 0.005	0.946
Chills, *n* (%)	4 (6.1%)	8 (4.3%)	χ^2^ = 0.312	0.577
LUTS worsening, *n* (%)	8 (12.1%)	28 (15.2%)	χ^2^ = 0.378	0.539
Duration of LUTS worsening (days), median (range)	10 (7–10)	10 (1–30)	Z = −0.173	0.863
Post-biopsy QoL, median (range)	2 (0–6)	1 (0–6)	Z = −2.733	0.006

Abbreviations: IPSS, International Prostate Symptom Score; QoL, quality of life; LUTS, lower urinary tract symptoms. *n*: Concordant prophylaxis, *n* = 66; non-concordant prophylaxis, *n* = 184.

## Data Availability

The data presented in this study are available on reasonable request from the corresponding author due to privacy and ethical restrictions. De-identified data can be provided upon request.

## Abbreviations

The following abbreviations are used in this manuscript:

BPH	benign prostatic hyperplasia
IPSS	International Prostate Symptom Score
IIEF-5	International Index of Erectile Function-5
LUTS	lower urinary tract symptoms
PCa	prostate cancer
QoL	quality of life
TRUS	transrectal ultrasound
